# Differential response of silencing *HvIcy2* barley plants against *Magnaporthe oryzae* infection and light deprivation

**DOI:** 10.1186/s12870-018-1560-6

**Published:** 2018-12-06

**Authors:** Blanca Velasco-Arroyo, Manuel Martinez, Isabel Diaz, Mercedes Diaz-Mendoza

**Affiliations:** 1 Centro de Biotecnologia y Genomica de Plantas (CBGP, UPM-INIA), Universidad Politecnica de Madrid (UPM) - Instituto Nacional de Investigacion y Tecnologia Agraria y Alimentaria (INIA), Campus Montegancedo UPM, 28223 Madrid, Pozuelo de Alarcon Spain; 2 Departamento de Biotecnologia-Biologia Vegetal, Escuela Tecnica Superior de Ingenieria Agronomica, Alimentaria y de Biosistemas, UPM, 28040 Madrid, Spain

**Keywords:** Cystatin, Biotic-abiotic stress, Proteolysis, Plant defense

## Abstract

**Background:**

Phytocystatins (PhyCys) act as endogenous regulators of cysteine proteases (CysProt) involved in various physiological processes. Besides, PhyCys are involved in plant reactions to abiotic stresses like drought or darkness and have been used as effective molecules against different pests and pathogens. The barley PhyCys-CysProt system is considered a model of protease-inhibitor regulation of protein turnover. Thirteen barley cystatins (HvCPI-1 to HvCPI-13) have been previously identified and characterized. Among them HvCPI-2 has been shown to have a relevant role in plant responses to pathogens and pests, as well as in the plant response to drought.

**Results:**

The present work explores the multiple role of this barley PhyCys in response to both, biotic and abiotic stresses, focusing on the impact of silencing this gene. *HvIcy-2* silencing lines behave differentially against the phytopathogenic fungus *Magnaporthe oryzae* and a light deprivation treatment. The induced expression of *HvIcy-2* by the fungal stress correlated to a higher susceptibility of silencing *HvIcy-2* plants. In contrast, a reduction in the expression of *HvIcy-2* and in the cathepsin-L and -B like activities in the silencing *HvIcy-2* plants was not accompanied by apparent phenotypical differences with control plants in response to light deprivation.

**Conclusion:**

These results highlight the specificity of PhyCys in the responses to diverse external prompts as well as the complexity of the regulatory events leading to the response to a particular stress. The mechanism of regulation of these stress responses seems to be focused in maintaining the balance of CysProt and PhyCys levels, which is crucial for the modulation of physiological processes induced by biotic or abiotic stresses.

**Electronic supplementary material:**

The online version of this article (10.1186/s12870-018-1560-6) contains supplementary material, which is available to authorized users.

## Background

Among plant protease inhibitors, the cysteine protease (CysProt) inhibitors called phytocystatins (PhyCys) have been largely studied since they participate in numerous physiological processes. The complexity of PhyCys at genomic and structural levels, with multiple members and specific expression patterns and affinity to CysProt, indicates a great diversity of functions [1–4]. PhyCys act as endogenous regulators of protein turnover in many physiological processes, such as plant growth and development, ripening, accumulation and mobilization of storage proteins, and programmed cell death, [2, 5–7]. PhyCys are also involved in plant reactions to abiotic stresses like drought or darkness [5, 8–13, 14] and senescence processes [15]. They have been shown to be up-regulated under water deprivation [16–19], but conversely a reduction in their expression has been also documented after drought stress [14, 20]. Enhanced drought tolerance in transgenic *Arabidopsis thaliana*, *Glycine max* and *Malus domestica* plants has been reported owing to the over-expression of PhyCys [21, 22]. PhyCys respond to other abiotic stresses like extreme variation of temperature and their over-expression make the plant more tolerant [23, 24]. Likewise, the over-expression of two cystatins, AtCYSa and AtCYSb, increased the resistance to a combination of abiotic stresses like drought, salt stress, cold and oxidative stress in Arabidopsis [9].

PhyCys superfamily is one of the most studied inducible defenses of the plant, and PhyCys has been used as effective molecules against different pests and pathogens [25–31]. Recombinant PhyCys have been shown to inhibit the activity of digestive proteases from many herbivores, affecting their development and reproduction when pests feed in artificial diets containing the recombinant PhyCys or in transgenic plants overexpressing a PhyCys gene [3, 4]. Likewise, several publications reported the induction of PhyCys in plants mediated by fungal infection [32, 33]. Recombinant PhyCys have been able to affect the in vitro growth of some phytopathogenic fungi [34, 35]. Transgenic approaches have also contributed to understand how plants over-expressing or silencing PhyCys genes respond to attacks by pathogens. Maize plants silencing the cystatin-9 gene had a reduced infection by *Ustilago maydis*, indicating that this PhyCys suppresses host immunity by inhibition of apoplastic cysteine proteases [32]. Conversely, tomato plants overexpressing the cystatin TaMDC1 showed high levels of resistance against *Alternaria alternata* and elevated tolerance against *Botrytis cinerea* [36]. Although the mechanism by which PhyCys inhibit fungal growth has not been yet elucidated, it has been suggested to be related to inhibition of fungal cysteine proteases [37–39] but it could also implicate changes in the permeability of fungal membrane [40]. In addition, PhyCys may act as stabilizing fusion partners for recombinant protein production in plants [41–43].

Barley (*Hordeum vulgare* L.) represents a good model to study the implication of cystatins in responses to biotic and abiotic stresses, given the comprehensive knowledge of its CysProt and PhyCys families. In previous works, thirteen cystatins (HvCPI-1 to HvCPI-13) have been identified and characterized [1, 2]. They have a role in response to abiotic stresses, in defense to biotic stresses and also participate in endogenous plant processes. Their function of defense against pests has been determined by their competence to inhibit the activity of insects and acari digestive proteases, using non-natural diets or plants stably transformed with barley PhyCys genes [4, 28–30, 44]. Three cystatins from barley (HvCPI-1, HvCPI-2 and HvCPI-6) and the mutated variant HvCPI-1 C68 → G have been transgenically expressed in barley, Arabidopsis, potato, tomato and maize to determine how they affect insect and mite performance [28, 29, 45, 46]. However, lesser is known about the in vivo barley PhyCys effects on pathogens. HvCPI-6 from barley inhibited the in vitro growth of some phytopathogenic fungi, including *Magnaporthe oryzae*, but Arabidopsis plants over-expressing this PhyCys showed no differences in fungal and bacteria resistance levels in comparison to non-transformed plants [47]. On the other hand, the participation of barley cystatin family members in abiotic stresses responses has been associated to a specific role modulating the degradative activity of endogenous proteases [12, 48]. The expression of barley PhyCys HvCPI-3-6 and 8–9 was induced by darkness [12], and HvCPI-2 together to HvCPI-4 showed the highest level of expression after water deprivation in barley leaves [48].

HvCPI-2 has been shown to have a relevant role in plant responses to biotic stresses, since earlier work performed by our group showed in vitro inhibition of the fungal growth of some phytopathogenic fungi like *M. oryzae* [47] as well as in vitro inhibitory activity of the cathepsin L- and B-like activities of several phytophagous arthropods [4]. In addition, recent work reported that tomato plants overexpressing this cystatin affect the performance of the lepidopteran *Tuta absoluta* [45]. *HvIcy-2* gene has been also analyzed under abiotic stresses like darkness and drought. While *HvIcy-2* gene is notably up-regulated under drought conditions [48] no differences were observed after light deprivation treatments [12]. An enhanced tolerance to drought at initial growing stages, together to a stay-green phenotype at the final stages of the plant life cycle was also observed in silenced HvCPI-2 lines [48]. To further explore the multiple roles of this barley PhyCys in response to both, biotic and abiotic stresses, and its effects on the cysteine proteinase-proteinase inhibitor system, we have analyzed in the current work phenotypical and molecular responses of *HvIcy-2* silencing lines to a fungal stress or to a light deprivation treatment.

## Results

### Expression of *HvIcy-2* is modified in barley leaves during the response to elicitors and *M. oryzae* treatments

**Fig. 1:**
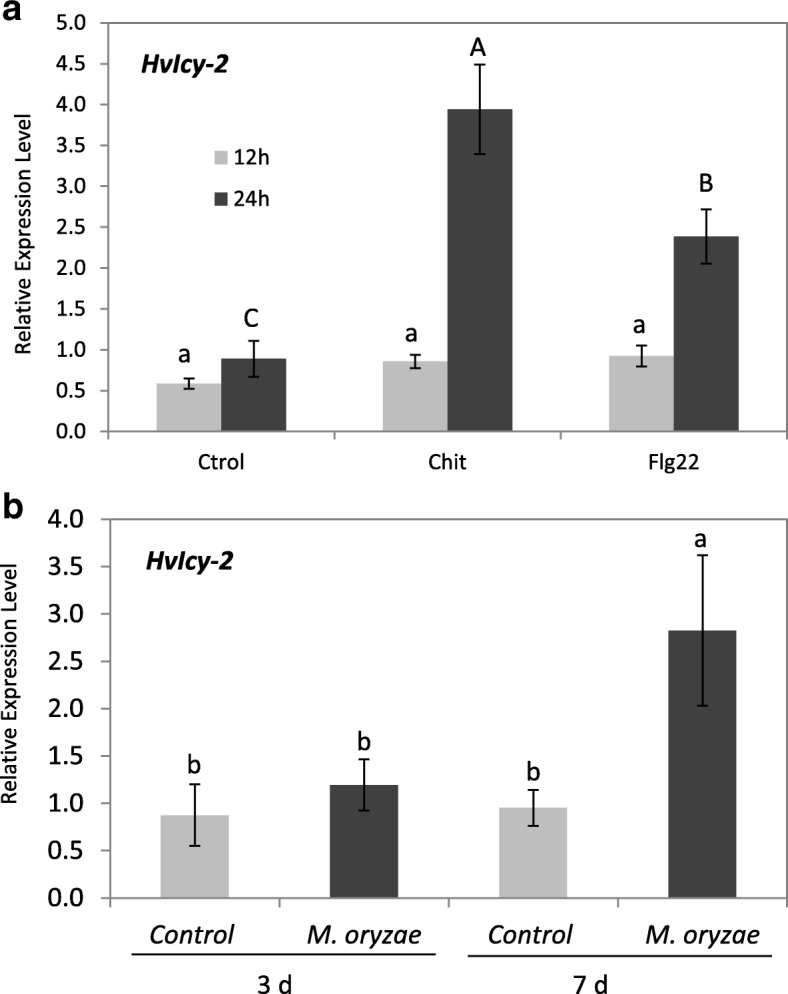
mRNA expression levels of barley *HvIcy-2* gene (**a**) after 12 (light grey) and 24 h (dark grey) of elicitor treatments in leaves with chitosan (Chit), flagellin (Flg22), and controls (Ctrol), and (**b**) barley wild type plants after *M. oryzae* infection at three (3 d) or seven (7 d) days after treatment from leaves of infected (dark grey) or non-infected (light grey) plants. Data were determined by RT-qPCR and expressed as relative mRNA levels of *HvIcy-2* gene normalized to barley *cyclophilin* mRNA content. Data are means ± standard error of at least three independent analyses. Different letters indicate significant differences (*P* < 0.05, One-Way ANOVA Student Newman-Keuls test)

The in vitro inhibition of the fungal growth of some phytopathogenic fungus like *M. oryzae* points to a relevant role for HvCPI-2 in plant responses to pathogens, [47]. To check if the expression of *HvIcy-2* was up-regulated upon biotic stresses, elicitors known to activate plant defense mechanisms against bacterial and fungal were selected [49]. For bacterial response treatments, the elicitor flg22, a sequence of 22-amino acid of the conserved N-terminal portion of bacterial flagellin, was tested. For fungal response, the effect of the elicitor chitosan, a cell wall of fungi structural element, was also tried. Using mRNA quantification, the expression patterns of *HvIcy-2* in response to elicitor treatments was analyzed and a molecular characterization was performed (Fig. 1a). These results revealed that the *HvIcy-2* gene was significantly induced in treated leaf samples after 24 h of flg22 and chitosan treatments (Fig. 1a). Data were expressed as mRNA levels normalized to the *cyclophilin* gene constitutively active in barley.

Both elicitors induced the expression of *HvIcy-2* gene, and the highest levels were observed after chitosan treatment, therefore, the next approach was to evaluate the effects of a fungal pathogen, *M. oryzae*, in treated barley leaves. Results showed no differences at 3 dpi but after 7 dpi of *M. oryzae* infection the *HvIcy-2* gene was significantly up-regulated (Fig. 1b).

### Expression of barley *HvIcy-2* gene performs different under *M. oryzae* or darkness stresses in silencing KD Icy2 lines

Homozygous barley plants silencing the *HvIcy-2* gene (KD Icy 1318, 1322, 1390 and 1399 lines) have been used to carry out in vivo experiments to test the response of modified plants towards drought [50]. To know how the expression of *HvIcy-2* varies in response to different abiotic or biotic stresses, the same KD Icy2 lines were used to measure the messenger *HvIcy-2* levels upon *M. oryzae* or darkness treatments.

**Fig. 2:**
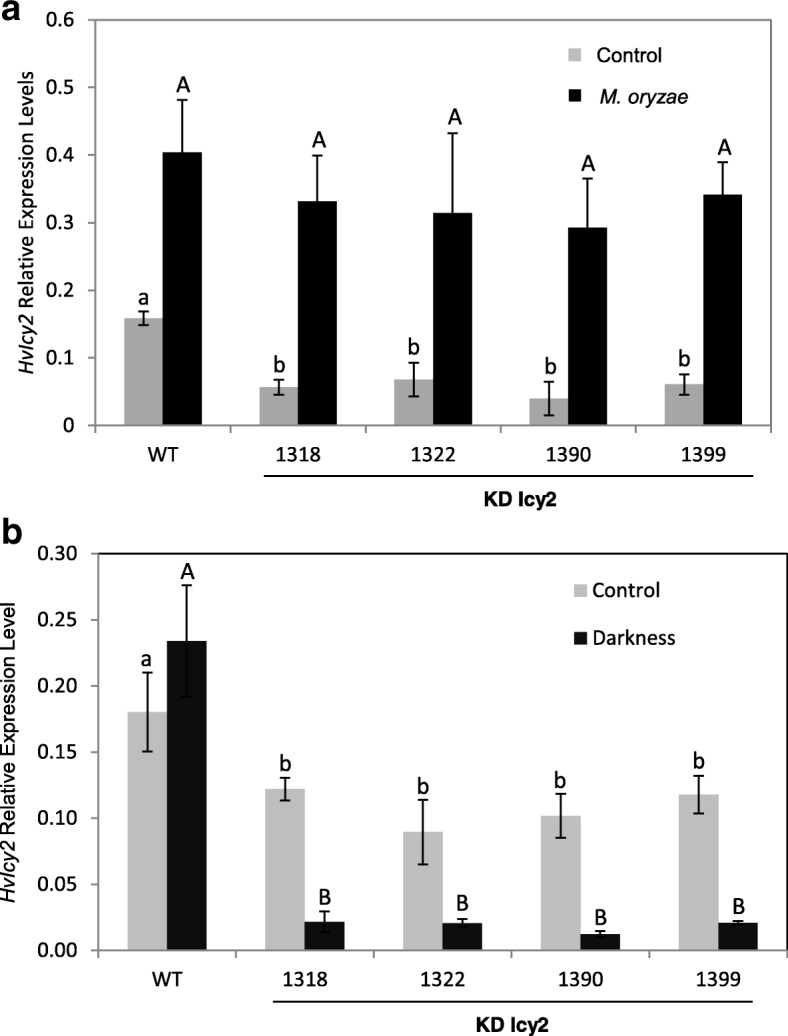
mRNA expression levels of barley *HvIcy-2* gene in transgenic *HvIcy2* silencing (KD Icy2, 1318, 1322, 1390 and 1399) lines, and wild type (WT) barley plants during darkness treatments (**a**) and *M. oryzae* infection (**b**), assayed by RT-qPCR. Total RNA was extracted from leaves after 14 days of darkness or 7 days of infection (dark grey) and non-treated/infected leaves (light grey). Data were expressed as mRNA levels of *HvIcy2* gene normalized to barley *cyclophilin* mRNA content. Data are means ± standard error of at least three independent analyses. Different letters indicate significant differences between lines. (*P* < 0.01, one-way ANOVA followed by Student Newman-Keuls test)

In Fig. 2a it can be observed a clear induction of the *HvIcy-2* gene after *M. oryzae* infection in *HvIcy-2* silencing barley lines, as well as wild type plants, when compared with control conditions. However, after light deprivation, the *HvIcy-2* gene in the transgenic lines rather than increase is down-regulated comparing to control conditions and comparing to WT, where no significant differences were observed (Fig. 2b).

### Transgenic barley *HvIcy-2* silencing lines show phenotypical differences after *M. oryzae* infection

**Fig. 3:**
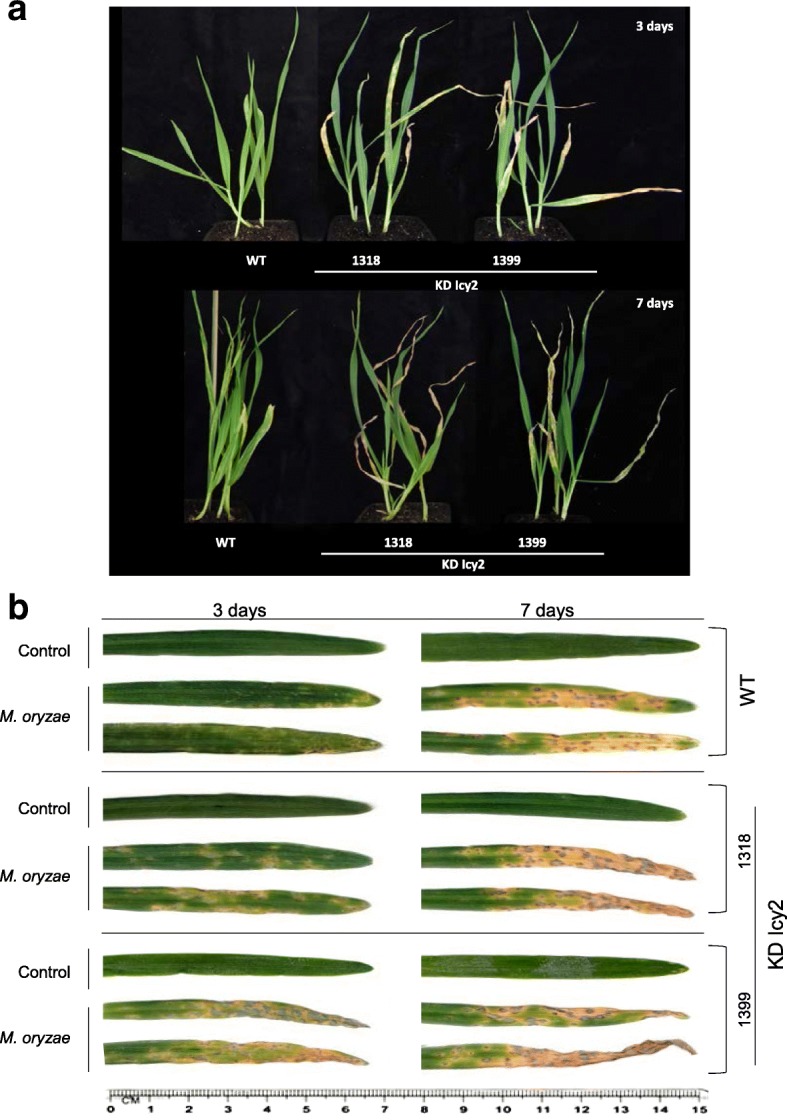
Images of the whole barley plant (**a**) and the oldest leaf (**b**) of transgenic *HvIcy2* silencing (KD Icy2, 1318 and 1399) and wild type (WT) barley lines after 3 days and 7 days of *M. oryzae* infection or non-infection as control

**Fig. 4:**
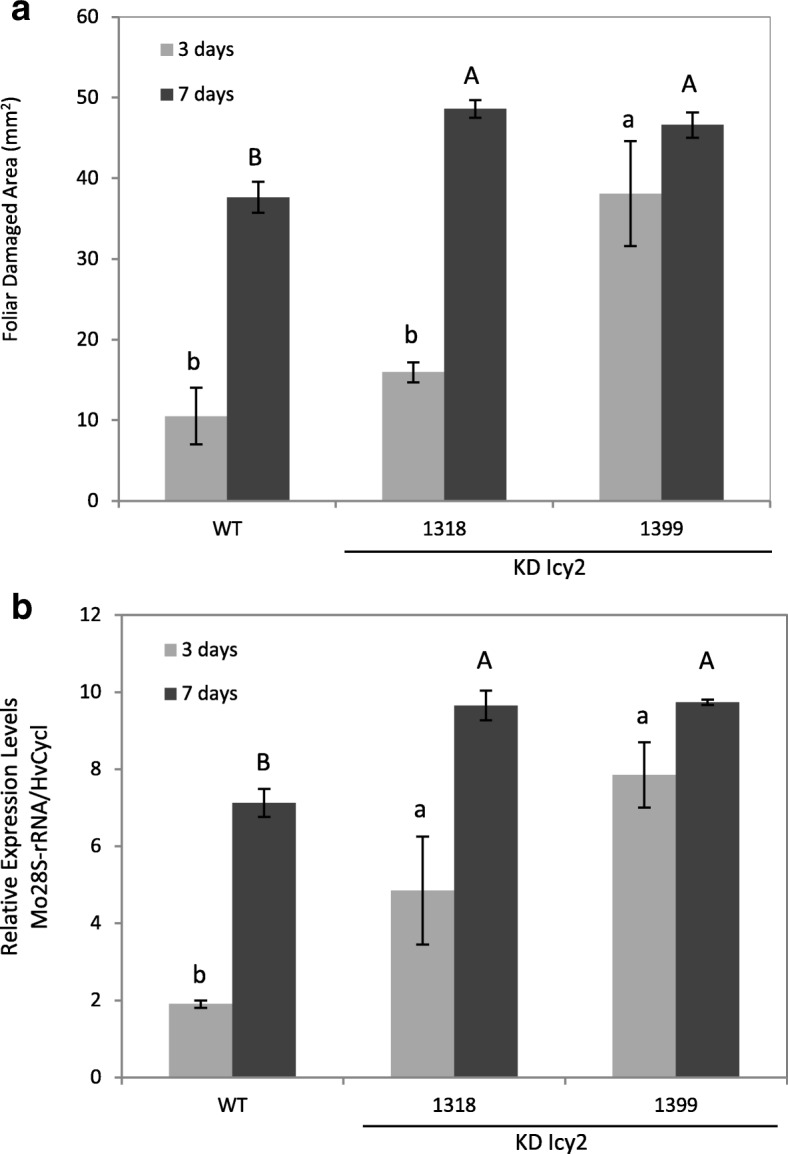
Quantification of leaf damage on barley transformed (KD Icy2 1318 and 1399) and non-transformed (WT) plants, after 3 (light grey) and 7 (dark grey) days of *M. oryzae* infection (**a**). Damage was measured as mm^2^ of injured foliar area and is represented as mean ± SE of seven old leaves measurements from seven independent plants per treatment. Effect of transgenic barley lines silencing the *HvIcy2* gene (KD Icy2 1318 and 1399) and wild type (WT) plants on *M. oryzae* performance (**b**). Quantification of *M. oryzae* small subunit of ribosomal RNA (*Mo28S-rRNA*) mRNA expression levels, at 3 (light grey) and 7 (dark grey) days after *M. oryzae* infection. Total RNA was extracted after infestation and data were expressed as mRNA levels normalized to barley cyclophilin mRNA content. Different letters indicate significant differences (P < 0.01, one-way ANOVA Student Newman-Keuls test)

*HvIcy-2* seems to have a role in plant responses to *M. oryzae* as it was up-regulated after infection treatments. Consequently, KD Icy2 barley plants were used to test the resistance or vulnerability of modified plants towards the attack of this fungus carrying out in vivo experiments. Comparing the behavior of wild type and transgenic plants after fungus infection, the participation of the *HvIcy-2* gene in the response to *M. oryzae* was analyzed (Fig. 3a-b). Further damage was observed in leaves from infected plants than those grown under control conditions, as was expected (Fig. 3b). Moreover, leaves from KD Icy2 lines, after 3 d of infection, showed higher damage than wild type (WT) barley plants (Fig. 3b). Subsequently, after 7 d of *M. oryzae* attack was more evident the highest vulnerability of KD Icy2 plants (Fig. 3b). Whole plant images highlight the reduced symptoms of damage in WT plants, which looked fewer susceptible to the fungus than KD Icy2 plants (Fig. 3a). In Fig. 4a the injured leaf area after infection was quantified in WT and transgenic infected lines, in order to compare the total damaged area. These results supported the previous observations. The WT plants, particularly at 7 days of *M. oryzae* infection, presented significantly lesser injured foliar area than KD Icy2 plants.

The existence of *M. oryzae* was registered by the measurement of the mRNA levels of the fungus small subunit of ribosomal RNA (*Mo28S-rRNA*), in order to analyze the effect of the modified barley plants on the fungus behavior (Fig. 4b). It was observed that at 3 and 7 days of infection a significantly greater amount of fungus mRNA was detected in KD Icy2 barley lines, which confirmed that these plants remained more vulnerable to *M. oryzae* than WT plants.

### Transgenic barley *HvIcy-2* silencing lines show small biochemical differences after light deprivation

**Fig. 5:**
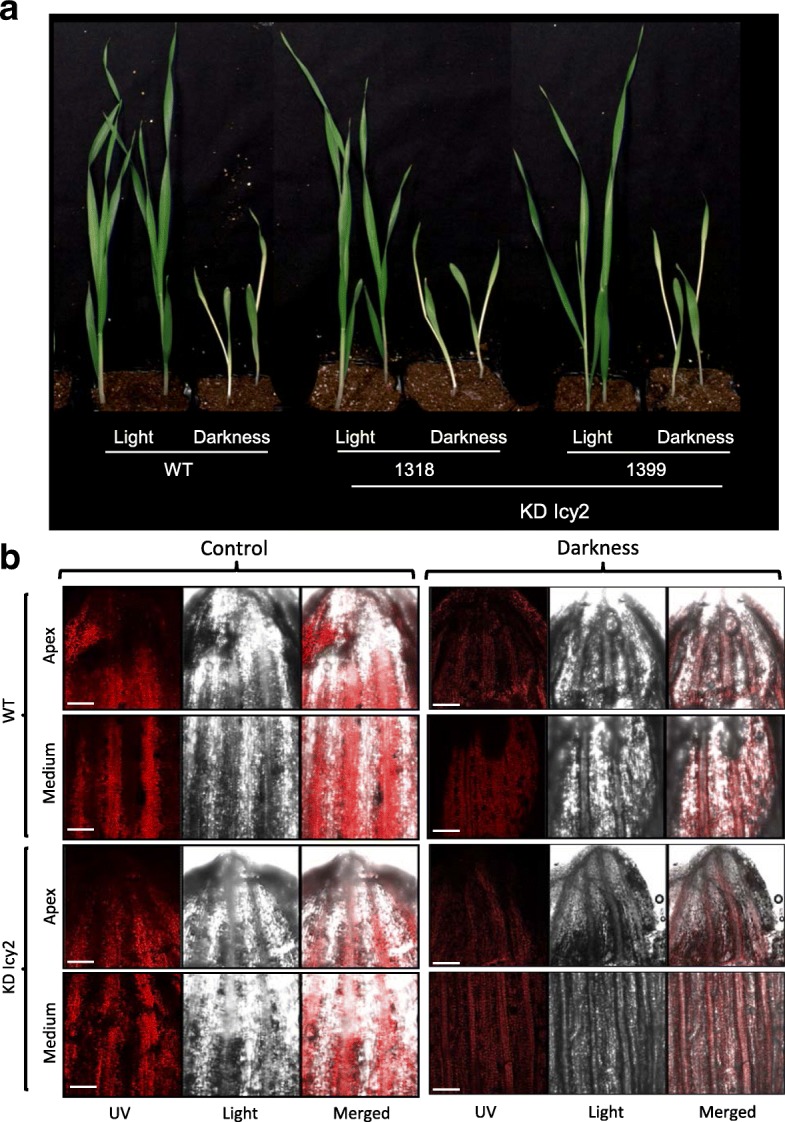
Phenotypes of WT and *HvIcy2* silencing (KD Icy2: 1318 and 1399 lines) barley plants grown in soil under continuous darkness or 16 h/8 h light/dark photoperiod for 14 days (**a**). Chlorophyll detection in the oldest leaf of transgenic and WT barley lines grown under darkness or 16 h/8 h photoperiod for 14 days (**b**). Leaf fragments from *HvIcy2* silencing (KD Icy2: 1318 line) and WT plants were collected and observed under a Leica SP8 confocal microscope using the laser excitation lines 633 nm to detect the red autofluorescence from the chlorophyll (UV). The same images were taken under light field conditions (light) and the fluorescence signal overlap is documented (merged). Leaves were cut in two fragments, corresponding to apical and medium-basal section of the leaf, respectively. Scale bar, 200 μm

**Fig. 6:**
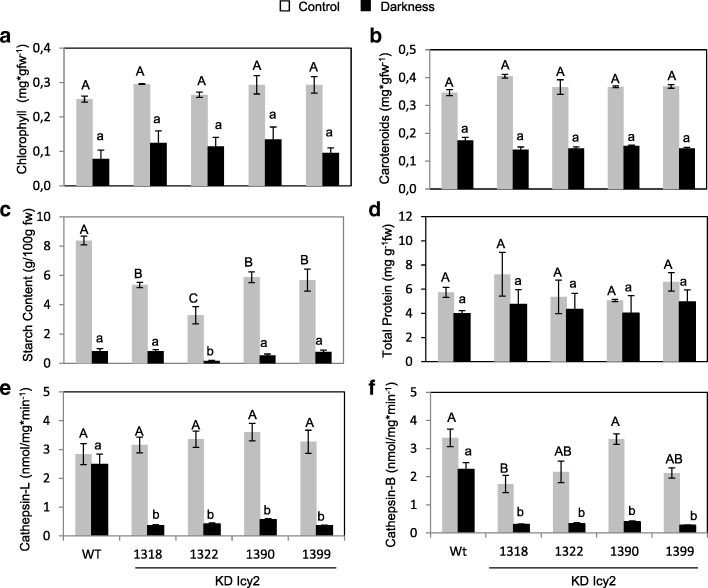
Photosynthetic pigments, chlorophyll (**a**) and carotenoids (**b**), content referred in mg of pigment per gram of initial fresh weight. Starch content (**c**) measured as grams of transformed starch per 100 g of fresh weight. Total protein content (**d**) referred in mg of total soluble protein per gram of initial fresh weight. Proteolytic activities with specific substrates to be degraded by cathepsin L/F-like (**e**) and B-like (**f**). *HvIcy2* silencing (KD Icy2: 1318, 1322, 1390, 1399 lines) and wild type (WT) barley plants were grown in soil under continuous darkness or 16 h/8 h photoperiod for 14 days. Data are means ± standard error of at least three independent analyses. Different letters indicate significant differences between lines. (*P* < 0.05, one-way ANOVA followed by Student Newman-Keuls test)

Once corroborated the role of *HvIcy-2* in the response to a biotic stress, we decided to further analyze the consequences of *HvIcy-2* repression after darkness treatment. After 14 days of darkness treatment, no phenotypical differences were observed in KD Icy2 silencing barley lines compared to WT (Fig. 5a). The chlorophyll content of the oldest leaf was observed by detecting its autofluorescence under a confocal microscope (Fig. 5b). The highest chlorophyll fluorescence was found in the medium segment of the leaf in both, transgenic and WT plants, in control conditions. A similar fluorescence emission was detected in apex and medium in KD Icy2 lines when compared to WT in darkness conditions. Comparable patterns were also found in leaf tissues structures observed under bright field grown under control or darkness treatments (Fig. 5b). Photosynthetic pigments were also analyzed in entire aerial biomass of the plants after 14 days of darkness vs control conditions. However, significant differences were not detected between transgenic and WT samples (Fig. 6a and b). The chlorophyll content of KD Icy2 plants was similar in comparison to WT, and a reduction in the total amount of chlorophyll was observed in all light deprived-stressed plants (Fig. 6a). This result supports chlorophyll auto-fluorescence observations (Fig. 5b). For the quantification of carotenoids, similar results were found in all lines (Fig. 6b). The starch and protein contents of the leaves were also quantified (Fig. 6c and d). As expected, starch accumulation was strongly reduced in all 14 days darkness-treated leaves. Although KD Icy2 transgenic leaves grown under photoperiod had significantly lower starch than WT leaves, no remarkable differences were detected between transgenic and WT samples under darkness conditions (Fig. 6c). Differing, no significant differences in protein content were found in KD Icy2 lines and nontransgenic lines when grown under photoperiod or light deprivation conditions (Fig. 6d). Finally, using specific substrates, the cathepsin L- and B-like proteolytic activities of KD Icy2 and WT plants grown in the darkness or under control conditions were determined. When plants were grown under light conditions, no significant differences on the cathepsin L-like activity were detected between transgenic and WT lines (Fig. 6e). Curiously, under darkness conditions the cathepsin L-like activity was significantly lower in KD Icy2 lines compared to WT plants (Fig. 6e). Comparable data resulted from the analysis of cathepsin B-like activity, which was significantly lower after light deprivation in KD Icy2 than in WT plants (Fig. 6f).

### Response to different stresses show molecular alterations with striking compensation effects at the transcriptional level

**Fig. 7:**
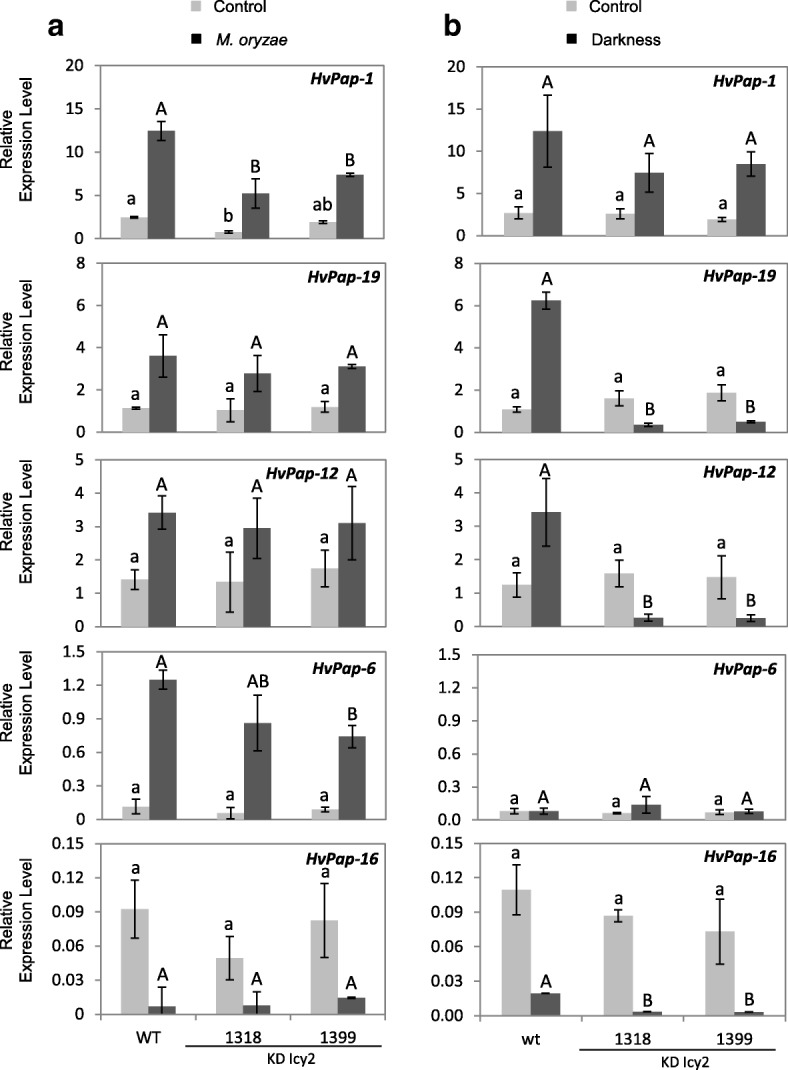
Messenger expression levels of C1A CysProt genes (*HvPap-1, − 19, − 12, − 6* and − *16*) in transgenic *HvIcy2* silencing (KD Icy2, 1318 and 1399) lines, and wild type (WT) barley plants assayed by RT-qPCR. (**a**) Total RNA was extracted from leaves after 7 days of *M. oryzae* infection (dark grey) and non-infected leaves (light grey). (**b**) Total RNA was extracted from leaves of WT and KD Icy2 barley plants grown in soil under continuous darkness (dark grey) or with 16 h/8 h photoperiod (light grey) for 14 days. Data were expressed as mRNA levels of C1A CysProt genes normalized to barley *cyclophilin* mRNA content. Data are means ± standard error of at least three independent analyses. Different letters indicate significant differences between lines. (*P* < 0.05, one-way ANOVA followed by Student Newman-Keuls test)

Variations in the transcript content of PhyCys may have an effect on the expression of their protease targets [48, 50]. Thus, the expression patterns for several CysProt belonging to different C1A subgroups (F-, L-, H-, and B-like cathepsins) were analysed by RT-qPCR under *M. oryzae* attack or light deprivation treatments (Fig. 7). Transcripts of *HvPap-1* CysProt gene increased in stressed plants independently of the treatment, *M. oryzae* infection or darkness, with *HvPap-1* levels being higher in WT than KD Icy2 after *M. oryzae* treatment (Fig. 7). Similarly, *HvPap-19*, *HvPap-12* and *HvPap-6* CysProt genes increase after *M. oryzae* infection in both, wild type and KD Icy2 plants (Fig. 7a). However, after light deprivation treatments, *HvPap-19* and *HvPap-12* genes were up-regulated in wild type and down-regulated in KD Icy2 when compared to control conditions and the expression of *HvPap-6* gene did no change after light deprivation treatment in any line (Fig. 7a and b). *HvPap-16 *showed a similar pattern of expression after both stress treatments, being greater down-regulated after darkness in KD Icy2 lines than in wild type (Fig. 7b). Based on these results, the variability in plant responses against different stress treatments may underlie the specificity of CysProt genes involved in the response as well as compensation effects on proteolytic-related proteins at the transcriptional level caused by the alteration in the expression of a PhyCys.

## Discussion

The participation of cystatin family members in biotic and abiotic stresses responses has been widely described [3]. Their participation is addressed to a precise regulation in which protease inhibitors have a specific role modulating the degradative activity of endogenous proteases as well as pest or pathogen proteases. However, the relevance of the role of an individual cystatin on several stresses has not been conveniently addressed. Based in previous results suggesting its participation in the response to biotic and abiotic stresses, the barley HvCPI-2 cystatin was selected to further explore the functional role of a cystatin against multiple threats [51]. Earlier work performed by our group showed in vitro inhibitory activity of HvCPI-2 on the cathepsin L- and B-like activities of several phytophagous arthropods [4], as well as in vitro inhibition of the fungal growth of some phytopathogenic fungus like *M. oryzae* [47]. The induction of PhyCys in plants mediated by fungal infection has been reported [33, 52], and recombinant PhyCys have been able to affect the growth of some phytopathogenic fungi in vitro [35, 40], highlighting the importance of PhyCys in response to pathogens. Likewise, transcriptional induction of *HvIcy-2* after elicitor and *M. oryzae* treatments further support a protective role of HvCPI-2 against pathogen attack. According to this, transgenic barley *HvIcy-2* silencing lines remained more susceptible to the fungus attack than the wild type lines. Although *HvIcy-2* expression was not significantly different in transgenic lines after fungus treatment when compared to wild type, we still found some increase of foliage area damage in silenced fungus-infected plants. This may be due to that the decreased expression of *HvIcy-2* gene in transgenic lines previous to the infection is enough to lesser protect the plant of the fungus attack. In other words, the lower level of *HvIcy-2* gene in transgenic lines is responsible of the higher susceptibility of these plants to the fungus attack. Even though the plant tries to overexpress this gene after the attack, it is not enough in time and quantity to be protected at the same level of wild type plants. Therefore, we suggest that *HvIcy-2* may have a role in fungal protection. Our results are in accordance with those from [36] that showed high levels of resistance against *A. alternata* and elevated tolerance against *B. cinerea* in tomato plants overexpressing the cystatin TaMDC1. On the contrary, [52] using transgenic plants silencing the cystatin-9 gene observed a reduced infection by *U. maydis*, but they argued that this PhyCys suppresses host immunity by inhibition of apoplastic cysteine proteases. Apart from this specific PhyCys-CysProt interaction, little is known on the physiological mechanism involved in the alteration of plant resistance/susceptibility when the expression of a cystatin is genetically modified. Two hypotheses should be taking into account. The first one is a direct toxic effect of the cystatin on the pathogen. The mechanism by which PhyCys inhibit fungi growth has not been yet elucidated. Several authors pointed out a direct inhibition of fungal cysteine proteases. The inhibitory activity of fungal growth exerted by recombinant cystatins from taro and amaranth was correlated with a partial inhibition of the cysteine protease activities of the fungal mycelium [37–39]. In contrast, other studies have indicated that variants of cystatins from barley and sesame lacking cysteine protease inhibitory activity had comparable ability to inhibit mycelial growth or spore germination of several phytopathogenic fungi. This suggests an alternative mechanism of action that could be associated to changes in the permeability of the fungal membrane [40, 53]. The second hypothesis is supported by the observed transcriptional reprogramming triggered by a genetic modification. Barley plants over-expressing the *HvIcy-6* gene had a down-regulation in the expression of photosynthetic related genes suggesting a primed state for a faster defense response [46]. Besides, tomato plants over-expressing *HvIcy-2* showed a higher expression of a tomato wound-induced serine protease inhibitor (*PIN2*) [45]. In any case, further research needs to be done to understand how plants over-expressing or silencing PhyCys genes respond to attacks by pathogens. *HvIcy-2* gene has been also analyzed under abiotic stresses. While *HvIcy-2* gene is notably up-regulated under drought conditions [48] no differences were observed after light deprivation treatments [12]. However, an unresponsive expression pattern does not mean absence of physiological role. To elucidate if HvCPI-2 activity affects darkness response, silencing *HvIcy-2* plants are a key tool. Interestingly, the expression of *HvIcy-2* decreased in silencing lines after dark treatment, but this lower *HvIcy-2* expression was accompanied to a decrease in cathepsin activities. Besides, these changes were not translated to biochemical or phenotypical differences. This striking behavior may be due to a transcriptional reprogramming of PhyCys expression to compensate *HvIcy-2* silencing. Most of the responses to abiotic stresses are a consequence of the direct inhibition of plant protease targets [13], and different PhyCys may participate in this inhibition. For instance, the expression of barley PhyCys *HvIcy-3-6* and *8–9* was induced by darkness [12] and *HvIcy-2* together to *HvIcy-4* showed the highest level of expression after water deprivation in barley leaves [48]. Mutual compensating expression of *HvIcy-2* and *HvIcy-4* genes in silencing lines after water deprivation supports a cooperative role of these two cystatins [48]. Likewise, transcriptional changes in PhyCys expression may be accompanied to transcriptional variations in the expression of their CysProt targets. In fact, an opposite effect of darkness on the transcript levels of *HvPap-12* and *-19* between control and silenced lines was found. This effect could partially explain the lower cathepsin activity showed by silencing *HvIcy-2* lines after darkness treatment and supports that responses to different stresses are accompanied to molecular alterations with striking compensation effects at the transcriptional level.

## Conclusions

In this study, it has been shown how transgenic barley *HvIcy-2* silencing lines perform different under biotic or abiotic stresses. Results highlight the specificity of PhyCys in the responses to diverse external prompts as well as the complexity of the regulatory events leading to the response to a particular stress. *HvIcy-2* silencing lines not only behave differentially against biotic or abiotic stresses, but also against two abiotic stresses like drought and light deprivation. The mechanism of regulation of these stress responses seems to be focused in maintaining the balance of CysProt and cystatins accumulation levels, which is crucial for the regulation of the physiological processes induced by biotic or abiotic stresses. Thus, HvCPI-2 may be participating directly on the stress, which would explain the highest susceptibility of KD Icy2 barley plants to the fungal attack, or a transcriptional reprogramming of the expression of different PhyCys and CysProt may be occurring to compensate *HvIcy-2* silencing, which would explain the low cysteine protease activity showed by silencing *HvIcy-2* lines after darkness treatment. Further research needs to be done to decipher the putative in vivo roles of cystatins and CysProt, and to deepen on the existing knowledge about the proteolytic events underlying responses to different stresses.

## Methods

### Plant material and growth conditions

Source of Plants: Barley plants of *H. vulgare* cv. Golden Promise were used. Barley transgenic lines silencing the *HvIcy-2* gene (KD Icy2) were generated as described in [50]. Grains of wild type and transgenic barley were germinated in soil and grown as described in [50], and the homozygous transgenic barley lines for the inserted constructions were correspondingly validated.

The plant specimens were taking from seeds of barley plants of *Hordeum vulgare* spring type cv. Golden Promise provided by the IPK Gatersleben, Plant Reproductive Biology Group. Barley transgenic lines silencing the barley *Icy-2* gene (KD Icy2) were generated also in collaboration with the IPK Gatersleben, Plant Reproductive Biology Group.

The fungal isolate used in this study for infection assays was the *Magnaporthe oryzae* wild-type strain Guy11 [54], kindly provided by Dr. Sesma, CBGP-UPM-INIA, Madrid. Plants and fungus used in our study complied with institutional, national, or international guidelines.

### Real-time RT-qPCR analysis

Total RNA was extracted from frozen barley leaves by the phenol/chloroform method and digested with DNase as described in [55]. cDNAs were synthesized from 2 μg of RNA using High Reverse Transcription kit (Applied Biosystems) following the manufacturer’s instructions. RT-qPCR analyses were performed using the SYBR Green detection system and expression levels of the *M. oryzae* small subunit of ribosomal RNA (*Mo28S-rRNA*) were quantified as described in [49]. Quantification was standardized to barley cyclophilin (*HvCycl* gene) mRNA levels following [12]. The primers used are shown in Additional file 1: Table S1.

### Protein quantification and protease activities

Total protein was extracted from treated and control leaves as described in [56], and quantified according to the method of [57], using the bovine serum albumin as a standard. Protease activities were assayed as described in [56], by measuring the hydrolysis of substrates containing the AMC (7-amino-4-methyl coumarin) fluorophore carried out in microliter plate format.

### Photosynthetic pigment measurements and starch quantification

Chlorophyll *a* and *b*, and total carotenoids (xanthophylls and carotenes) were extracted from leaves and pigment content was calculated as described in [56]. To detect the red auto-fluorescence from the chlorophyll, the oldest leaf of each transgenic and wild type plant was observed under a Leica SP8 confocal microscope (Leica, Wetzlar, Germany) with 633 nm laser excitation line. Thirty mg of fresh leaves from wild type and transgenic barley lines were used for total starch quantification using the STA20 Kit (Sigma) as described in [56].

### Elicitor treatments

The flagellin peptide (flg22) with amino acid sequence QRLSTGSRINSAKDDAAGLQIA (AnaSpec laboratories) and chitosan purified from crab shells (Sigma Aldrich) were used for elicitor treatments as described in [49]. Elicitor solutions were applied over 7 days old wild type barley leaves as a foliar spray and plants were further incubated at the same growth conditions described earlier. Three independent experiments were performed and 3 pots of 3 plants each were used per treatment. Barley leaves were harvested after 12 and 24 h of elicitor treatments, frozen into liquid nitrogen and stored at − 80 °C for further analysis.

### *M. oryzae* infections

Seven days old barley plants of wild type and transgenic lines silencing the *HvIcy-2* gene were infected with the fungus *M. oryzae* as described in [49]. The *M. oryzae* wild type strain Guy11 kindly provided by Dr. Sesma (CBGP-UPM-INIA, Madrid) was the fungal isolate used. The infection assays were performed as described in [58] by spray inoculations using an airbrush nebulizer compressor in whole plant leaves. Three independent experiments were performed and seven plants were used per treatment. Barley leaves were harvested after 3 and 7 days of fungus treatment, frozen into liquid nitrogen and stored at − 80 °C for further analysis.

### Damage quantification assays

*M. oryzae* damage observed on barley leaves were scanned using a HP Scanjet 5590 Digital Flatbed Scanner and foliar damage was analyzed as described in [49] using the Fiji-ImageJ software [59]. Three independent experiments were performed and seven replicates were analyzed. Data were represented as foliar damaged area (mm^2^) mean ± SE of seven measurements.

### Data analysis

Statistical differences among treatments or lines were analyzed by one-way ANOVA followed by studentized range distribution of Student Newman-Keuls (SNK) multiple comparison test performed using the soft R Project (v.3.1.2) package. At least three biological replicates as well as three technical replicates were performed for all the experiments.

## Additional file

Additional file 1: **Table S1.** List of primers. (DOCX 17 kb)

## Abbreviations

AMC: 7-amino-4-methyl coumarin
CysProt: Cysteine proteases
flg22: Flagellin peptide 22
PhyCys: Phytocystatins
RT-qPCR: Real-time quantitative PCR analysis
SNK: Student Newman-Keuls multiple comparison test
