# Supplementary material for: Differential response of silencing HvIcy2 barley plants against Magnaporthe oryzae infection and light deprivation
Source: BMC Plant Biol. 2018 Dec 6;18:337. doi: 10.1186/s12870-018-1560-6 (PMC6282322; doi:10.1186/s12870-018-1560-6)
Supplement: Supplementary file 1 — Table S1. List of primers. (DOCX 17 kb) [file 12870_2018_1560_MOESM1_ESM.docx]

Additional file 1: Table S1 Primer sequences used for the amplification of genes by RT-qPCR assays. *HvCycl* (barley cyclophilin), *HvIcy-2* gene (barley cystatin-2), *HvPap-1* gene (barley cathepsin F-like protease), *HvPap-6,* and *HvPap-16* genes (barley cathepsin L-like proteases), *HvPap-12* gene (barley cathepsin H-like protease), *HvPap-19* gene (barley cathepsin B-like protease) and *Mo28S-rRNA* gene (*Magnaporthe oryzae* small subunit of ribosomal RNA).

| **Genes** | **Primers** |
| --- | --- |
| *HvCycl* | forward: 5’-TCCACCGGAGAGGAAGTACAGT-3’  reverse: 5’-AATGTGCTCAGAGATGCAAGGA-3’ |
| *HvIcy-2* | forward: 5´-TCCTGGAGTCGATCTTGGTTTC-3´  reverse: 5´-CAAGCATACTGTTGCGGCTTC-3´ |
| *HvPap-1* | forward: 5´-TCCTGGAGTCGATCTTTGGTTTC-3’  reverse: 5´-CAAGCATACTGTTGCGGCTTC-3’ |
| *HvPap-6* | forward: 5´-TGCAATTGACGGCAAGAAGA-3´  reverse: 5´-TGGATCACCAGGTGATCATTTG-3´ |
| *HvPap-12* | forward: 5´-ATGTGCGCTATTGCTACCTGC-3´  reverse: 5´-CACCTTATTCATGTCTGGCGAA-3´ |
| *HvPap-16* | forward: 5´-CTGGATCGGTAAGAACTCGTGG-3´  reverse: 5´-TGATGGAGGTGCCATCATATGA-3´ |
| *HvPap-19* | forward: 5´-CACCTTATTCATGTCTGGCGAA-3´  reverse: 5´-TGCCCGCTTAATTTGACAGG-3´ |
| *Mo28S-rRNA* | forward: 5´-TACGAGAGGAACCGCTCATTCAGATAATT-3´  reverse: 5´-TCAGCAGATCGTAACGATAAAGCTACTC-3´ |
